# Plant Extracts to Alleviating Heat Stress in Dairy Cows

**DOI:** 10.3390/ani13182831

**Published:** 2023-09-06

**Authors:** Yongmei Guo, Li Li, Sumei Yan, Binlin Shi

**Affiliations:** Key Laboratory of Animal Nutrition and Feed Science at University of Inner Mongolia Autonomous Region, College of Animal Science, Inner Mongolia Agricultural University, Hohhot 010018, China

**Keywords:** dairy cows, heat load, natural active components, regulatory mechanism

## Abstract

**Simple Summary:**

Climate change and environmental heat stress are important challenges for the future of the dairy industry, as these environmental factors can reduce the productive and reproductive performance of dairy cows. Therefore, relieving the heat stress of dairy cows is an important priority for the dairy industry. Plant extracts have advantages in safety, efficiency, low toxic side effects (if correctly selected) and residue, thus, can significantly aid in alleviating heat stress in dairy cows. This paper reviews the effects of some plant extracts on alleviating heat stress in dairy cows and their possible regulatory mechanisms. The aim is to develop applicable strategies using plant extracts to alleviate heat stress in dairy cows.

**Abstract:**

Heat stress (HS) in cows is a critical issue in the dairy industry. Dairy cows accumulate heat from body metabolism, along with that imposed by air temperature, humidity, air flow and solar radiation. HS in animals can occur during hot and humid summers when the ambient temperature is extremely high. Dairy cows have relatively high feed intakes and metabolic heat production and are thus susceptible to HS, leading to reductions in feed intake, lower milk yield, affected milk quality, reduced animal health and even shortening the productive lifespan of cows. Therefore, alleviating HS is a top priority for the dairy industry. Suitable plant extracts have advantages in safety, efficiency and few toxic side effects or residues for applications to alleviate HS in dairy cows. This paper reviews the effects of some plant extract products on alleviating HS in dairy cows and briefly discusses their possible mechanisms of action.

## 1. Introduction

Climate change and heat stress (HS) are important challenges facing the dairy industry, as heat stress detrimentally affects the productive and reproductive performances of dairy cows [1]. HS causes a decrease in dry matter intake (DMI) and oxidative distress in dairy cows [2], resulting in a decrease in milk yield [3] and impairment of the animals’ health. Farmers can adopt physical cooling methods, such as shading, fans and moisture spraying, to alleviate HS, but the investment is usually costly and adds an additional economic burden. Spraying can lead to raised environmental humidity, and this can lead to more frequent occurrences of foot and limb diseases and mastitis [4]. These heat-stress-associated economic losses can be significant. The ban on antibiotic use in feed has led to more and more studies being focused on using plant extracts to alleviate heat stress in dairy cows. Plant extract refers to those naturally bioactive compounds extracted from plant materials, that have shown specific physiological effects on animals [5]. Research has shown that plant extracts can alleviate HS in farm animals by enhancing their immune competence and heat resistance [6], mitigating the detrimental effects on body temperature and productivity [7], improving their antioxidant status and promoting immune functions [8]. In addition, plant extracts have the advantages of safety, high efficiency, and few toxic side effects (if correctly selected) or residues when correctly identified [9]. Therefore, plant extracts could have a significant application in alleviating HS in dairy cows.

## 2. Detrimental Effects of Heat Stress on Dairy Cows

HS is a series of non-specific defense reactions that occur when cows are subjected to temperatures that exceed their thermoregulatory capacity [10]. Currently, the level of HS in cows is assessed using a temperature–humidity index (THI), rectal temperature and respiratory rate. The optimal environmental temperature for Holstein cows is between 0 °C and 20 °C [11]. When the ambient temperature exceeds 25 °C and the THI is greater than 68, the cows will experience HS [12]. When the THI exceeds 72, cows will enter a mild HS state, leading to a decrease in feed intake and nutrient absorption [13]. Feed intake is the foundation for the growth and development of animals, and feed intake decreases with increasing ambient temperatures [14]. For example, a decrease in DMI of 10 kg/d will reduce milk yield by 12 kg/d in lactating Holstein cows [15]. In addition, HS reduces rumination and resting time, together with longer standing time and increased panting [16,17]. These changes affect the healthy and productive status of cows, as indicated by physiological and biochemical parameters, including hormone profiles, oxidative status, immune functions, rumen fermentation patterns, and declining cow productivity [18].

### 2.1. Effect of Heat Stress on Lactation Performance

During hot summers when both temperature and humidity are high, the metabolic heat produced by cows cannot be released in a timely manner, resulting in a raised body temperature, leading to HS [19]. This is particularly so in lactating cows with high feed intakes and thus high rates of metabolic heat production to meet the nutrient requirements for milk production. When ambient temperatures and humidity are high, the accumulated heat will further exacerbate HS in cows. Research in eight major milk-producing provinces in China has shown that hot summer environmental conditions are associated with significantly lower cow feed intake and milk yield compared with cooler seasons [3,20]. Mild and moderate HS results in milk yield declines of 0.13 kg for every unit increase in the THI [21]. However, the decrease in DMI under HS explained only 35% of the decrease in milk protein [22]. Recent studies have shown that HS induces a reduction in milk protein precursor synthesis and apoptosis of bovine mammary epithelial cells (BMEC), leading to a redistribution of the precursors being used excessively for gluconeogenesis to compensate for the energy deficit caused by HS [23,24]. In addition, HS increases the susceptibility of cows to mastitis. Mastitis-causing pathogens are shed from the infected mammary gland quarters and thus contribute to an increased risk of intra-mammary gland infections [25]. The causative udder pathogens are predominantly *staphylococci*, *streptococci* and *coliform* species [26]. A recent study has also shown that a rise in THI results in *Staphylococcus* shedding at relatively high concentrations [27]. HS reduces milk protein concentration and yield, which may be due to the inhibition of the synthesis of milk proteins, especially the synthesis of α and β caseins [28,29]. In addition, HS significantly reduces the nutritional value of milk by reducing phospholipids, with a declining abundance of phosphatidylethanolamine, phosphatidylserine, phosphatidylcholine, lysophosphatidylcholine and glucosylceramide in milk [30].

### 2.2. Effect of Heat Stress on Rumen Microbes

Temperature and other internal environmental factors affect the metabolism of microorganisms in the rumen. Rumen microorganisms are most active in a narrow temperature range of 39.0 to 39.5 °C, and microbial diversity is significantly reduced in conditions of high environmental temperature and humidity [31]. *Prevotella* is an important family of Bacteroidetes and is usually more abundant in the rumen of high-yield cows [32]. During HS, the relative abundance of *Prevotella* is significantly reduced [31], along with milk fat and protein concentrations, as the THI increases [33]. This indicates that HS affects milk fat and protein concentrations through the abundance of rumen *Prevotella*. The species of Bacteroidetes in the rumen of cows increased with HS, which significantly affected the decomposition of ingested feed. An increase in the ratio of Bacteroidetes to Firmicutes is associated with a reduction in fat mobilization [34] to meet the energy needs of lactating cows. HS significantly decreases the ratio of Bacteroidetes to Firmicutes [35], likely resulting in an inadequate energy supply for milk production.

### 2.3. Effects of Heat Stress on Antioxidant Status and Immune Functions

HS can cause oxidative distress, which can contribute to dysfunctional inflammatory responses in cows [36]. In in vitro studies, HS treatments significantly decreased cell viability and reduced the activities of catalase (CAT), superoxide dismutase (SOD), and total antioxidant capacity (T-AOC), but elevated intracellular levels of malondialdehyde (MDA) and reactive oxygen species (ROS) [37]. Previous studies demonstrated that maternal HS can also have carry-over effects on immune function, particularly increasing the neutrophil-lymphocyte ratio [38]. Long-term HS can increase the plasma concentration of interleukin-6 (IL-6), causing inflammation in dairy cows [39]. A study on the effects of HS during late pregnancy in cows reported that HS resulted in increased expression of IL-1β and IL-1RA mRNA in maternal serum [40].

## 3. The Role of Plant Extracts in Alleviating Heat Stress in Dairy Cows

Previous studies have shown that dietary supplementation with bupleurum extract [7] and honeysuckle extract [8] can alleviate HS in dairy cows. Currently, plant extracts containing polyphenols and flavonoids are widely used in livestock [41] to alleviate HS [42], enhance immune functions [43], increase feed intake [44] and improve product quality [45].

### 3.1. Polyphenols

Plant polyphenols are polyhydroxy compounds mainly found in the roots, bark and leaves of plants; these include phenolic acids, flavonoids, 1,2-stilbene compounds, and lignins [46]. Some studies have found that polyphenols can up-regulate enzymatic (e.g., SOD, glutathione peroxidase (GPx), CAT) and non-enzymatic (e.g., glutathione, GSH) antioxidant defense systems [47], improve endothelial functions and prevent the development of cardiovascular diseases [48]. Procyanidins, a subclass of a natural polyphenol compound, are widely present in plant fruits, vegetables and nuts [49]. Among various isomers of procyanidins, procyanidin B2 (PB2) is the main dimer with highly powerful antioxidant activity [50]. PB2 can protect BMEC from heat-induced oxidative stress, significantly enhancing CAT, SOD and T-AOC levels and reducing MDA and ROS production, as well as IL-1β concentration [37]. Zeng et al. suggested that *Astragalus* polysaccharides influence hormone profiles in the serum of heat-stressed dairy cows and regulate glucose and amino acid metabolism pathways. They also identified twenty potential biomarkers, including up-regulated glucose-1-phosphate, glutamine, glycerol-1-phosphate, glycine, lysine, pyrophosphoric acid, putrescine, tryptophan, tyrosine and down-regulated 2-picolinic acid, 3-aminoisobutyric acid, alanine, γ-aminobutyric acid, glucose, sugar alcohol, nicotinamide, norvaline and phenylacetic acid [51]. Resveratrol is a natural polyphenol compound widely found in *Polygonum cuspidatum*, peanut species and other plants that has various biological activities, such as being anti-inflammatory and regulating energy metabolism [52]. Kra et al. demonstrated that resveratrol has an antioxidant effect by reducing MDA concentration in bovine dedifferentiated adipocyte-derived progeny cells and may induce adipose lipolysis and reduce lipogenesis under HS conditions in vitro [53].

### 3.2. Flavonoids

Plant flavones are secondary metabolites of plants, derived from the phenylpropane metabolic pathway, and have ameliorative effects in dairy cows suffering from HS [54]. Plant flavonoids are natural antioxidants that release hydrogen ions and scavenge oxygen-free radicals by binding to reactive oxygen species, thereby improving cellular viability [55]. Recent studies have shown that feeding bamboo leaf flavonoids to dairy cows increases the activity of BMEC [56] and reduces oxidative damage, resulting in increased milk yield (1.08 kg/d), milk fat (0.20 kg/d) and milk protein (0.02 kg/d) [57]. In vitro studies found that an addition of 75 ug/mL of alfalfa flavonoids to the culture medium increased BMEC and GPx activity while decreasing MDA content and lactate dehydrogenase (LDH) activity under HS conditions [58]. Baicalin, a flavone glycoside, has pharmacological effects, including antioxidant activity and scavenging oxygen-free radicals [59]. Phytoflavonoids can also improve the specific immune function of organisms by increasing antibody levels and cell apoptosis activity. Feeding soy flavonoids to lactating cows suffering from HS increases the levels of immunoglobulin G (IgG), interferon-α (IFN-α) and IL-2 in the blood [60]. Dihydromyricetin has cyto-protective effects on heat-stressed BMEC by reducing mitochondrial membrane depolarization and dysfunction, bax and caspase 3 activity, modulating oxidative enzyme activity, and reducing ROS production and apoptosis [61]. Adding puerarin to heat-stressed bovine Sertoli cells (BSCs) suppresses ROS and MDA production and enhances SOD, CAT and GPx activity [62]. Treating heat-stressed BSCs with baicalin reduces cell apoptosis via the modulation of the cell survival rate through the Fas/FasL pathway and up-regulation of HSP72 expression [63].

### 3.3. Other Plant Extracts

In addition to the aforementioned polyphenols and flavonoids, there are other plant compounds that function to alleviate HS and improve antioxidant and immune functions in animals. Capsaicin is an active component of *Capsicum annuum* that promotes feed intake and relieves HS in cows [64]. The main bioactive compound in *Radix bupleurum* extract is saponin. Feeding heat-stressed lactating cows with *bupleurum* extract increases feed intake and milk yield by 9.09% and 8.23%, respectively [7]. Betaine is an organic osmolyte sourced from sugar beet, and supplementing with betaine increased milk yield (1.40 kg/d), fat yield (0.05 kg/d) and protein yield (0.04 kg/d) in grazing dairy cows that were experiencing HS [65]. In another study of heat-stressed dairy cows, using a supplement of 15 g/day of betaine in stall feeding increased feed intake, milk yield and milk protein concentration by 5.27%, 2.63% and 4.34%, respectively [66]. Feeding herbal mixtures was shown to increase milk yield from 16.3 kg/d to 16.9 kg/d in heat-stressed cows [67]. Supplementation with a citrus extract decreased somatic cell counts in the milk of heat-stressed dairy cows [68]. Berberine, a small herbal molecule, is the main active component of Chinese herbal medicine Huanglian (*Coptis chinensis*), and its antipyretic effect has been shown to regulate HSP70 [69].

## 4. Potential Regulatory Mechanisms of Plant Extracts in Alleviating Heat Stress in Dairy Cows

### 4.1. Alleviating Heat Stress by Lowering Cortisol

When domestic livestock experience HS, the adrenal gland plays a central role, with cortisol secretion being an important biological indicator of HS [70]. The HS stimulates the hypothalamic–pituitary–adrenal axis (HPA) in cattle through internal and external receptors, increasing the secretion of cortisol [71]. The increased secretion of cortisol can suppress the immune function of macrophages and accelerate lymphocyte apoptosis, thereby suppressing host immune responses and leading to increased morbidity [72]. Meanwhile, elevated inflammatory cytokines affect cortisol signaling and contribute to the increased release of cortisol, leading to abnormal HPA axis function [73]. A study demonstrated that HS increased serum cortisol levels in lactating cows [74]. Jujube powder contains jujube polysaccharides, flavonoids, saponins and other bioactive substances that have been shown to decrease serum cortisol concentrations in heat-stressed chickens [75]. *Agastache rugosus* contains an essential oil that reduces serum cortisol in beef cattle suffering from HS, thus alleviating some of the effects of HS [76]. Peng et al. reported that the supplementation of 400 mg puerarin per kg of diet reduced serum cortisol concentrations in chronically stressed beef cattle [77]. In rats, resveratrol treatment inhibited cortisol production from liver microsomal vesicles in a concentration-dependent relationship [42,78]. The foregoing results suggest that active substances extracted from plants can reduce cortisol concentrations in animals subjected to HS and, therefore, can alleviate some of the detrimental effects of stress.

### 4.2. Inhibiting the HSP70/NF-κB Signaling Pathway

The heat shock response is an adaptive regulatory response of cells to high temperatures, as indicated by the heat shock transcription factor (HSF) and heat shock proteins (HSPs), which are extremely sensitive to HS. When HS occurs, HSF-1 initiates the transcriptional process and up-regulates the expression of HSPs, thus repairing the misfolded proteins and resisting HS damage. Heat-stress-induced ROS production and calcium ions are involved in the phosphorylation of HSF1 and the regulation of HSP expression [79]. The serum HSF, HSP27, HSP70 and HSP90 concentrations are significantly increased in cows undergoing HS [80]. Among these proteins, HSP70 is an important molecular chaperone and the most abundant and functional HSP in the HSP family, and its expression is usually used as a biological marker for HS. Alderman et al. reported that the HSP70 concentration was closely related to changes in ambient temperature and humidity and played an important role in the thermostability of organisms [81]. When animals experience HS, HSP70 can improve the degree of cellular tolerance to thermal damage, maintain normal physiological functions and cellular metabolism, and improve overall cell survival [82]. The relative abundance of HSP70 mRNA is gradually up-regulated during heat exposure [83]. HSP70 binds to the cell membrane as a cytokine, causing calcium inward flow and activating nuclear transcription factor-κB (NF-κB), while up-regulating the expression of tumor necrosis factor-α (TNF-α) [84]. The NF-κB signaling pathway is an important pathway closely related to antioxidation and anti-inflammatory activity and is mediated by toll-like receptor 4 (TLR4). Oxidative stress promotes the glycosylation modification of NF-κB/p65, and this then activates the NF-κB signaling pathway to promote the production of down-stream inflammatory cytokines, such as IL-1β, IL-6, TNF-α [85]. Some plant extracts can modulate these pathways. For example, berberine inhibits the gene expression of HSP70 and TNF-α by suppressing the binding of the TATAbox binding protein (TBP) to the “TATAbox”, which ultimately leads to a decrease in body temperature [64]. Mogroside-rich extracts inhibit NF-κB and HSP70 expression, thus protecting against heat-stress-induced intestinal damage by reducing inflammation and oxidative stress [86]. IL-6 is a pro-inflammatory cytokine that activates the NF-κB pathway, and gintonin-enriched extract can produce antipyretic effects by decreasing the level of IL-6 in muscle cells [87,88]. Quercetin and kaempferol protect Sertoli cells from HS by suppressing the levels of p-NF-κB-p65, which can activate NF-κB [89].

### 4.3. Activation of Nuclear Factor-Associated Factor E2 Pathway Alleviates Heat Stress

High temperatures stimulate the production of ROS, resulting in an imbalance between oxidants and the antioxidant defense system [90]. Nrf2 is an important transcription factor that regulates cellular resistance to oxidative stress [91] and is essential for cellular resistance to external stimuli and the maintenance of intracellular redox homeostasis [92]. Under normal physiological conditions, Nrf2 is bound to its cytoplasmic blocker, Kelch-like epichlorohydrin-associated protein-1 (Keap1), with Nrf2 activity tightly regulated by Keap1 [93]. Upon stress stimulation in vivo, the sensor Keap1 receives signals and changes its conformation to dissociate from Nrf2. Nrf2 then enters the nucleus and binds to the macrophage activating factor (Maf), forming a heterodimer. The heterodimer recognizes and binds to the promoter of the antioxidant response element, thereby promoting the expression of down-stream protective phase II detoxification enzymes and antioxidant enzymes, including hemeoxygenase-1 (HO-1), CAT and SOD, thus improving the resistance of cells and tissues to oxidative damage caused by environmental heat or other factors [94]. Resveratrol can activate the Nrf2 signaling pathway, promote nuclear translocation of Nrf2 and increase its expression to improve antioxidant function, thereby protecting BMEC from oxidative stress induced by heating [95]. The addition of bamboo leaf flavonoids to cultured BMEC inhibits ROS production and reduces apoptosis while up-regulating the gene expression of Nrf2, HO-1, thioredoxin reductase 1 (TrxR1) and quinone oxidoreductase 1 (NQO1), thus protecting BMEC from heat stress-induced oxidative damage [96]. Baicalin, a flavonoid-containing compound, can reduce HS damage by inhibiting the expression of Keap1 and Nrf2 in the mouse uterus and regulating oxidase activity [97]. Baicalin reduces the expression of inflammatory cytokines by blocking the TLR4 signaling pathway and exerting antipyretic effects [98]. Choline protects BMEC from oxidative HS induced by inhibiting Nrf2 expression and nuclear translocation [99].

### 4.4. Mitigation of Heat Stress through AMPK/mTOR Signaling Pathway

HS not only reduces milk yield but also decreases milk quality, leading to “milk protein reduction syndrome” in dairy cows [100]. Components of milk proteins, such as the total casein and α and β casein contents, gradually decrease [101]. In vitro studies confirmed that HS reduces the gene expression of αs1-casein and β-casein in BMEC [102]. The mammalian target of the rapamycin (mTOR) signaling pathway is one of the major pathways for the synthesis of milk proteins, mainly via the regulation of protein translation. The mTOR up-stream signaling pathway is phosphatidylinositol-3 kinase/protein kinase B (PI3K-AKT), and activated PI3K acts on AKT. The activated AKT further acts directly on the mTOR pathway to phosphorylate the down-stream eukaryotic translation initiation factor 4E binding protein 1 (EIF-4EBP1) and ribosomal protein S6 kinase 1 (S6K1), thus promoting the synthesis of milk protein [103]. Studies have shown that HS can change the cytoskeleton structure of BMEC, inhibit the cell cycle, and significantly down-regulate the expression of genes involved in the mTOR signaling pathway (*RICTOR*, *PIK3* and *ELF2*) [104]. Amino acids (AA) are not only the precursors for the synthesis of milk protein, but they are also a special regulatory factor that regulates the proliferation of BMEC and the synthesis of casein by activating signal pathways in the cells [30]. Leucine (Leu) is one of the mTOR activators and the main regulator of mRNA in the production of proteins [105]. Activated transcription factor 4 up-regulates AA biosynthesis and indirectly induces the synthesis of Leu transporter by up-regulating transporter-related genes, mainly *SLC7A5*, which in turn activates mTOR [106]. HS decreases milk protein synthesis through its reduction in AA transporter carrier activity in enterocytes [107], resulting in a reduction in AA entering BMEC for milk protein synthesis. It has been shown that the addition of *Astragalus* polysaccharide as a feed supplement for heat-stressed cows can up-regulate Leu [51]. Bamboo leaf flavonoids up-regulate the expression of mTOR, S6K1 and EIF-4EBP1 genes and the expression of β-casein genes in BMEC [108]. As an important up-stream target of the mTOR signaling pathway, adenosine 5′-monophosphate (AMP)-activated protein kinase (AMPK) is an important energy transducer that directly or indirectly regulates the activity of mTOR complex 1 and is involved in multiple types of stress responses [109]. When ambient temperature increases from 18 °C to 30 °C, the activity of AMPK increases 9.1-fold and AMPK signaling molecules are activated in heat-stressed cows [110]. The activation of the AMPK signaling pathway inhibits the mTOR signaling pathway, resulting in a decrease in milk protein synthesis [111], leading to the “milk protein reduction syndrome” in cows. However, the use of plant extracts to regulate milk protein synthesis in heat-stressed cows through the AMPK/mTOR signaling pathway has not yet been reported and needs to be further studied.

## 5. Conclusions and Perspectives

**Figure 1:**
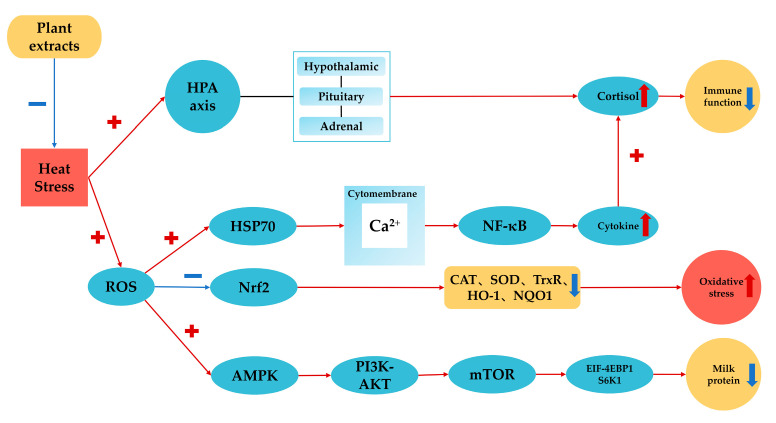
Potential regulatory mechanisms of plant extracts in alleviating heat stress in dairy cows.

Plant extracts have considerable potential in the alleviation of HS symptoms in dairy cows. Polysaccharides, polyphenols, flavonoids and other active ingredients in plant extracts can decrease serum cortisol and enhance antioxidant and immune functions, thus alleviating HS symptoms and improving milk production in heat-stressed lactating cows. However, the regulatory mechanisms are not fully understood due to the complexity of functional compounds in plant extracts. Thus, we proposed the following possible regulatory mechanisms: (1) the decrement of cortisol and alleviation of HS through the HPA axis; (2) the inhibition of HSP70 gene expression and blocking of other NF-κB signaling pathways; (3) the regulation of milk protein synthesis through changing the AMPK/mTOR signaling pathway and the regulation of gluconeogenesis/glycolysis and amino acid metabolism in heat-stressed cows. These potential mechanisms are summarized in Figure 1. The role of plant extracts in HS alleviation and corresponding regulatory mechanisms could vary depending on plant ingredients and natural plant variations. Further research should focus on a combination of field studies, laboratory analyses of functional compounds, and in vitro investigations of particular regulation mechanisms so that strategies to use plant extracts to alleviate HS in dairy cows can be developed with confidence.

## Data Availability

All data referred to in the manuscript are already published.
